# A study on the influencing factors of mental health of Chinese garden workers: a cross-sectional study

**DOI:** 10.1186/s12889-024-18025-8

**Published:** 2024-03-12

**Authors:** Yujin Xie, Yang Jiao, Lei Shi, Di Liu, Ying Liu, Zhen Tang, Weijun Gong, Hong Yu, Yuanshuo Ma

**Affiliations:** 1 Beijing Rehabilitation Hospital, Capital Medical University, Beijing, China; 2 School of Health Management, Guangzhou Medical University , Guangzhou, China; 3 School of Marxism, Harbin Medical University, Harbin, China; 4 School of Public Health, Hebei Medical University, Hebei, China

**Keywords:** Mental health, Job satisfaction, Stress, Anxiety, Depression, Garden workers

## Abstract

**Background:**

Occupational hazards occur in all walks of life. China’s horticulture industry is undergoing rapid development. However, the mental health of garden workers has not received much attention. This study investigates the mental health status and influencing factors of Chinese garden workers and provides a basis for promoting their mental health and ensuring the healthy development of Chinese horticulture.

**Methods:**

A cross-sectional survey of garden workers in Beijing was conducted from 10 July 2021 to 10 October 2021. A total of 3349 valid questionnaires were recovered, with an effective response rate of 95.69%. Descriptive statistical analysis was carried out on the demographic characteristics, job satisfaction, stress, anxiety, and depression of garden workers, and the influencing factors affecting the mental health of Chinese garden workers were found through a t-test, variance analysis, and ordinal multi-class logistic regression analysis.

**Results:**

Survey respondents were mostly male (54.4%) and under the age of 40 (64.1%). The anxiety and depression symptoms of the garden workers were moderate. Among staff members, 40.2% were in a normal state of stress. Gender, three meals on time, monthly income, and job satisfaction were the factors influencing stress, anxiety, and depression symptoms among garden workers.

**Conclusion:**

Compared to medical staff and other groups, the stress, anxiety, and depression symptoms of Chinese garden workers are severe. Gender, monthly income, and job satisfaction are important factors affecting their mental health. Managers should continuously improve the working environment of garden workers, provide salaries that match their positions, and improve their job recognition and satisfaction to reduce the impact of negative emotions on personal health.

## Introduction

Occupational injuries occur in people from all walks of life, including medical workers, construction workers, farmers, and gig workers [1–3]. Occupational injuries are classified into four main types: physical, chemical, biological, and psychosocial hazards. Recently, an increasing number of researchers have focused on psychosocial hazards associated with occupational injuries [4–7].

Garden workers experience various occupational hazards during their daily work. For example, this work may involve confined spaces, sloping or unstable surfaces, and physical harm caused to employees by the need to climb trees into branches [7]. A Danish cohort study found an association between pesticide exposure and Parkinson’s disease in gardeners [8]. A survey of 367 horticultural and arboriculture practitioners in Hong Kong, China, found that workers in such industries had a high rate of accidents at work, they were generally exposed to biological hazards and were less aware of chemical injuries [9]. Moreover, the occurrence of occupational hazards not only damages the health of individual gardeners but also causes loss of corporate profits and wastage of national medical resources [10].

Previous studies have examined more occupational hazards, such as physical hazards [7], biological hazards [9], and chemical hazards [8], suffered by garden workers, however, there are few studies on the mental health of garden workers. As a kind of occupational hazard, psychosocial hazards are widely present in all kinds of occupational groups, and they cannot be ignored. Anxiety, depression, and stress receive high social attention. Anxiety is a psychological and physiological state characterised by cognitive, physiological, and behavioural components, which is considered to be ‘anticipation of future danger or misfortune, accompanied by physical symptoms of restlessness or tension’ [11]. Depression is defined as an emotional disorder characterised by significant and prolonged mood swings, reduced behaviour, and loss of pleasure [12]. Stress is defined as a state of worry or mental tension caused by a difficult situation [13]. Previous research has shown that the incidences of anxiety, depression, and stress are high in certain occupational groups. For example, Nader Salari and others found that during the COVID-19, the prevalence of depression among nursing staff caring for patients was 24.3%, and the prevalence of anxiety was 25.8% [14], Naser Parizad found that the mean job stress of ICU nurses was ‘moderate’ [15]. Hamid Saeed et al. found that university teachers also experienced more severe anxiety and depression [16]. Scholars have also shown that 57% and 33% of farmers were classified as possible and possible cases for anxiety, respectively; the prospective recommendations for depression were 34% and 15% [17]. In response to this phenomenon, more scholars have conducted research on the factors influencing anxiety, depression, and stress. The research results showed that more psychological and social factors [18, 19] are associated with demographic characteristics [18], anxiety, depression, and stress in different groups. Therefore, several scholars have implemented intervention measures to reduce anxiety, depression, and stress in the relevant population [20–22]. Most studies have consistently found that interventions to prevent and promote mental health are cost-effective or cost-saving [14, 23]. Although previous researchers have conducted extensive research on the mental health of different populations, the studies related to garden workers remain limited.

Mental health affects physical health. Persistent negative emotions, such as stress, anxiety, and depression, can lead to a decline in the quality of an individual’s work [6], resulting in poor gardening and greening environments. A poor greening environment also negatively impacts physical and mental health [24, 25]. Poor mental health may also affect individual life satisfaction and quality of life [26, 27]. The absence of mental health leads to negative changes in physical health [28].. The formation of this vicious circle poses a serious threat to the healthy development of individuals and their families.

With the rapid development of Chinese society, urban, rural, and individual families have begun to pay attention to garden greening, which is also one of the evaluation criteria for liveable cities. This has also led to a growing demand for garden workers. However, their physical and mental health has not received sufficient attention worldwide. This study explores the correlation between the demographic characteristics (including job satisfaction) of Chinese frontline garden workers and their mental health (anxiety, depression, and stress) through cross-sectional research and analyses the psychological health status and reasons for this population. This can not only better understand the mental health of garden workers and promote their mental health but also provide a basis for ensuring the healthy development of China’s horticulture undertakings. It can also serve as a reference for other countries to study the physical and mental health of garden workers.

## Materials and methods

### Data collection

A cross-sectional study of garden workers in Beijing was conducted from 10 July 2021 to 10 October 2021. As a large city with a concentrated population, Beijing has several garden workers and high levels of urban greening. Its urbanisation level is higher than that of most other cities in China and comparable to that of developed countries. This is also the stage that cities in developing countries will experience in the future. Therefore, Beijing has a strong representation and research on the mental health of garden workers in this city can provide a reference for both developed and developing countries. The survey was conducted anonymously using an online questionnaire. To ensure that the investigation does not have a negative impact on the mental health of the garden workers, after the survey, garden workers were provided with intervention measures such as music therapy (sleep aid series, stress relief series, anxiety relief series, depression relief series, and calming music), relaxation, and stress reduction (meditation training, pleasing videos, relaxation training, and psychological balance training). Garden workers could choose any of these areas to regulate their emotions. Simultaneously, we ensure anonymity when completing the questionnaire for garden workers.

In general, the sample size is usually 5–10 times of the items [29, 30]. The number of questions in this survey is approximately 100, and the sample size is approximately 500–1000. Possible situations, such as incomplete questionnaires, incorrect questions, and sampling distribution during the questionnaire survey, were considered. A total of 3500 questionnaires were distributed. This study conducted a questionnaire survey by directly contacting the person in charge of the garden companies. Beijing has seven districts; five garden companies were randomly selected from each district. Using the probability sampling method, 10% of garden workers were selected from each garden company for a questionnaire survey. We sent a link to the online survey webpage to the participants via mobile phone and the gardeners answered in their free time. This survey method not only enables the manager of the questionnaire to monitor the collection of the questionnaire in real time but also effectively manages the data. The literature indicates that several researchers conducted studies using this survey method [31]. A total of 3500 garden workers participated in the formal survey to fill in the online questionnaire, of which 3349 were valid questionnaires; the questionnaire validity response rate was 95.69% (incomplete, incorrect, or online response time < 5 min of questionnaires were considered invalid).

The inclusion criteria for this study are: (1) aged over 18; (2) workers working in the field, such as flower arrangers, greeners, and horticulturists; (3) voluntary participation. The exclusion criteria are (1) garden workers who are unwilling to participate in the survey, and (2) other personnel who do not belong to the work category (such as drivers) (3). The family has experienced major changes or other events within one year before the investigation, which have a significant impact on their mental and psychological health; and (4) garden workers with a history of mental illness.

### Measurements

#### Demographic characteristics

The respondents’ demographic characteristics include gender, age, marital status, three meals on time, working years, and monthly income.

#### Depression anxiety stress scale (DASS)

Loivdband et al. compiled a self-assessment scale in 1995 to measure the severity of negative mood disorders (depression, anxiety, and stress), with 42 entries in the original edition [32]. Antony et al. retained seven entries for each dimension of the forgiveness table and reduced them to DASS-21 [33]. Each item was scored on a 4-point Likert scale (0 = not applicable to me at all, 1 = applicable to me to some extent or some time, 2 = applicable to me to a considerable extent, or most of the time applicable to me, 3 = very applicable to me, or most of the time applicable to me). The following cut-off values were used for each subscale [34]: Depression: Normal 0–4, Mild 5–6, Moderate 7–10, Severe 11–13, and Extremely Severe 14+; Anxiety: Normal 0–3, Mild 4–5, Moderate 7–10, Severe 11–13, and Extreme Severity 10+; and Stress: Normal 0–7, Mild 8–9, Moderate 10–12, Severe 13–16, and Extreme severity 17+.

The Chinese version of the DASS-21, developed by TAOK et al. (2001), has been tested as a reliable and effective tool in Hong Kong. It was first introduced and applied in 2010 among university students in the Chinese mainland [35]. In 2012, Wen et al. modified the Chinese version of the DASS-21 to make it more suitable for Chinese culture and assessed its reliability and validity in adults aged 18 years and older [36]. The items related to anxiety symptoms are 2, 4, 7, 9, 15, 19, 20; The items related to depressive symptoms are 3, 5, 10, 13, 16, 17, 21; The items related to stress symptoms are 1, 6, 8, 11, 12, 14, 18. This scale has been widely used in previous studies [37–39]. In this study, Cronbach’s alpha coefficient for this scale is 0.94, indicating good reliability.

### Job satisfaction scale

The Job Satisfaction Scale, developed by Brayfield and Rothe (1951), initially contained 18 items [40]. This study uses a simplified version of the job satisfaction scale with a total of six items, including the nature of the work, superiors, colleagues, income, promotion opportunities, and six aspects of the work situation, using the five-point Likert-scale, from 1 (extremely dissatisfied/totally disagree) to5 (very satisfied/totally agree); the higher the score, the higher the satisfaction of the respondents. The effectiveness and reliability of this scale have been demonstrated in previous studies [41]. The Chinese version of this scale has also been used in previous studies on Chinese people [42]. In this study, Cronbach’s alpha coefficient for this scale is 0.86, indicating good reliability.

### Data analysis

This study used SPSS 26.0 for statistical analysis. A descriptive statistical analysis of the demographic characteristics and job satisfaction of garden workers, as well as their anxiety, depression, and stress conditions, was conducted. A single-factor analysis of the relationship between the demographic characteristics, job satisfaction of the respondents, and their anxiety, depression, and stress conditions was performed using univariate analysis and an independent sample t-test. The meaningful factors were used as independent variables, and anxiety, depression, and stress conditions were used as dependent variables for ordered multi-classification logistic regression analysis to clarify the influencing factors of garden workers’ mental health, *P* < 0.05 was considered to be statistically significant.

## Results

**Fig. 1:**
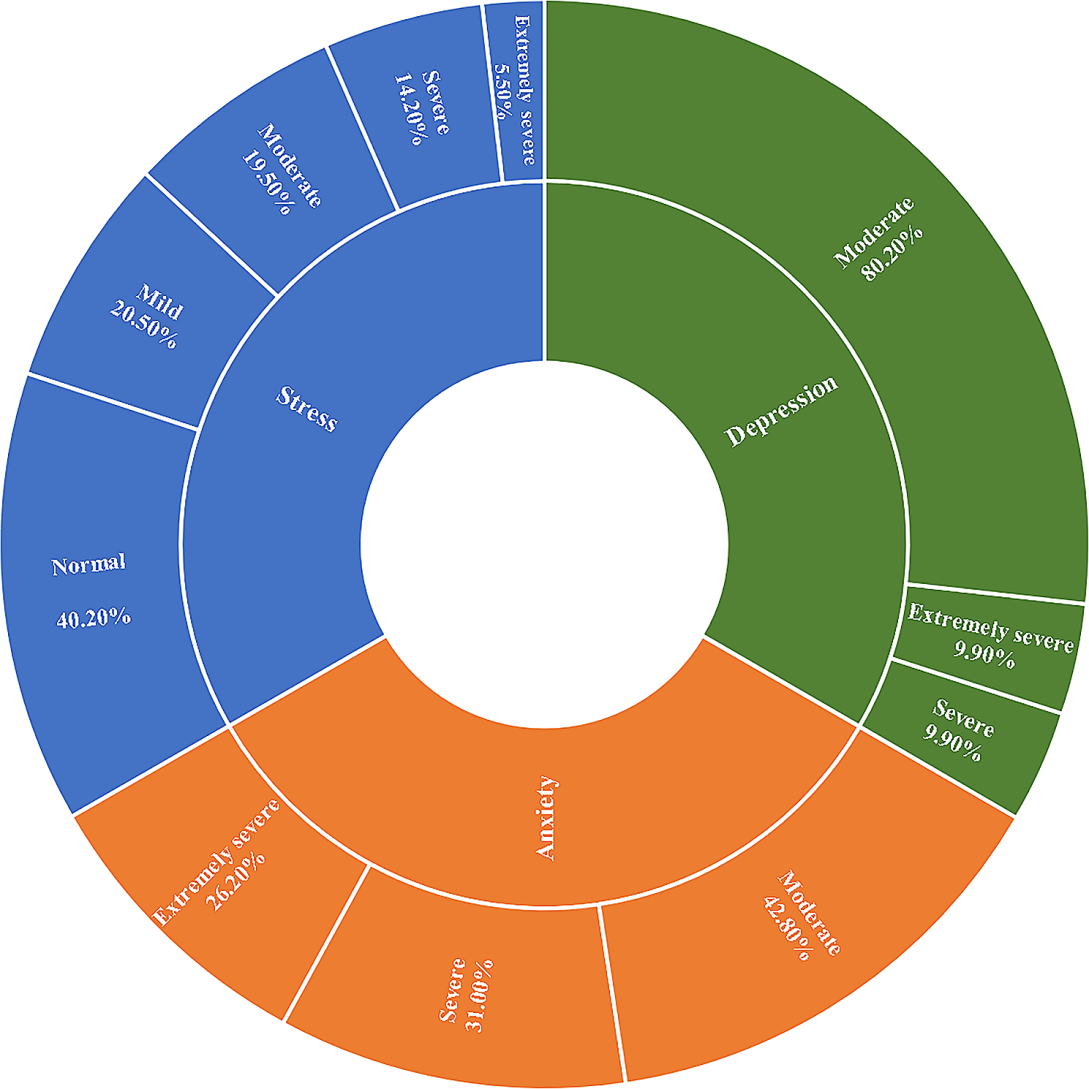
Prevalence of depression, anxiety and stress among Chinese garden workers

The actual number of people surveyed in this study was 3500, and the number of effective surveys was 3349, of which 1821 were men, accounting for 54.4%; 2146 people under the age of 40, accounting for 64.1%; 74.2% of the respondents were married; Most employees have worked for more than five years (62.3%), and 2087 employees have a monthly income of more than 5000 yuan, accounting for 62.3%; Garden workers have a low level of job satisfaction (22.04 ± 4.32) (Table 1). The anxiety and depression symptoms of garden workers were more serious, both in moderate and above; 57.2% had severe anxiety and 19.8% had severe depression. Among the participants, 40.2% were in a normal stress state. (Fig. 1).

**Table 1:** Demographic characteristics

Variables	N	Percent (%)
**Gender**		
Male	1821	54.4
Female	1528	45.6
**Age**		
≤ 30	935	27.9
31–40	1211	36.2
41–50	753	22.5
51–60	440	13.1
≥ 60	10	0.3
**Marital status**		
Married	2485	74.2
Single	765	22.8
Divorce	90	2.7
Widowed	9	0.3
**Three meals on time**		
All	2724	81.3
Breakfast is not allowed	322	9.6
Lunch is not allowed	53	1.6
Dinner is not allowed	171	5.1
Not at all	79	2.4
**Working years**		
≤ 1	145	4.3
2–5	1117	33.4
6–10	1043	31.1
≥ 11	1044	31.2
**Monthly income**		
≤ 1000	29	0.9
1001–2999	72	2.1
3000–4999	1161	34.7
≥ 5000	2087	62.3

The results of the one-factor analysis showed that the gender (T=-6.624, *P* < 0.001), age (F = 3.548, *P* < 0.001), marital status (F = 6.051, *P* < 0.001), whether they could eat three meals on time (F = 7.437, *P* < 0.001), monthly income (F = 22.851, *P* < 0.001), and job satisfaction (F = 3.032, *P* < 0.001) were factors that influenced depression. Gender (T=-2.799, *P* < 0.05), whether they could eat three meals on time (F = 8.936, *P* < 0.001), monthly income (F = 9.211, *P* < 0.001), and job satisfaction (F = 3.632, *P* < 0.001) were factors that influenced their anxiety state. Gender (T=-6.900, *P* < 0.001), age (F = 6.627, *P* < 0.001), marital status (F = 6.641, *P* < 0.001), whether they could eat three meals on time (F = 14.712, *P* < 0.001), monthly income (F = 12.001, *P* < 0.001), and job satisfaction (F = 4.005, *P* < 0.001) were the factors influencing their stress levels. (Table 2) This study controlled for related confounding factors through a single-factor analysis to avoid influencing the research results.

**Table 2:** Single factor analysis of garden workers

Variables	Depression(N/%)						Anxiety(N/%)						Stress(N/%)					
	Normal	Mild	Moderate	Severe	Extremely severe	F/T	Normal	Mild	Moderate	Severe	Extremely severe	F/T	Normal	Mild	Moderate	Severe	Extremely severe	F/T
Gender						-6.62***						-2.80*						-6.90***
Male	0/0.0	0/0.0	1531/45.7	161/48.6	129/39.0		0/0.0	0/0.0	796/23.8	598/17.9	427/12.8		791/23.6	388/11.6	367/11.0	208/6.2	67/2.0	
Female	0/0.0	0/0.0	1156/34.5	170/5.1	202/6.0		0/0.0	0/0.0	638/19.1	441/13.2	449/13.4		555/16.6	300/9.0	287/8.6	268/8.0	118/3.5	
Age						3.55***						1.65						6.63***
≤ 30	0/0.0	0/0.0	719/21.5	125/3.7	87/2.6		0/0.0	0/0.0	369/11.0	299/8.9	263/7.9		326/9.7	205/6.1	184/5.5	161/4.8	55/1.6	
31–40	0/0.0	0/0.0	983/29.4	116/3.5	105/3.1		0/0.0	0/0.0	518/15.5	394/11.8	302/9.0		469/14.0	237/7.1	255/7.6	178/5.3	65/1.9	
41–50	0/0.0	0/0.0	624/18.6	60/1.8	68/2.0		0/0.0	0/0.0	332/9.9	239/7.1	181/5.4		340/10.2	162/4.8	134/4.0	82/2.4	34/1.0	
51–60	0/0.0	0/0.0	343/10.2	27/0.8	70/2.1		0/0.0	0/0.0	203/6.1	115/3.4	122/3.6		200/6.0	80/2.4	77/2.3	52/1.6	31/0.9	
≥ 60	0/0.0	0/0.0	9/0.3	1/0.0	0/0.0		0/0.0	0/0.0	6/0.2	2/0.1	2/0.1		5/0.1	3/0.1	2/0.1	0/0.0	0/0.0	
Marital status						6.05***						1.94						6.64***
Married	0/0.0	0/0.0	2046/61.1	208/6.2	231/6.9		0/0.0	0/0.0	1096/32.7	756/22.6	633/25.5		1052/31.4	499/14.9	482/14.4	324/9.7	128/3.8	
Single	0/0.0	0/0.0	568/17.0	112/3.3	85/2.5		0/0.0	0/0.0	301/9.0	246/7.3	218/6.5		259/7.7	167/5.0	154/4.6	137/4.1	48/1.4	
Divorce	0/0.0	0/0.0	67/2.0	10/0.3	13/0.4		0/0.0	0/0.0	33/1.0	34/1.0	23/0.7		34/1.0	18/0.5	15/0.4	14/0.4	9/0.3	
Widowed	0/0.0	0/0.0	6/0.2	1/0.1	2/0.1		0/0.0	0/0.0	4/0.1	3/0.1	2/0.1		1/0.1	4/0.1	3/0.1	1/0.1	0/0.0	
Three meals on time						7.44***						8.94***						14.71***
All	0/0.0	0/0.0	2231	237	256		0/0.0	0/0.0	1215/36.3	843/25.2	666/19.2		1163/34.7	567/16.9	510/15.2	349/10.4	135/4.0	
Breakfast is not allowed	0/0.0	0/0.0	233/66.6	56/7.1	33/7.6		0/0.0	0/0.0	112/3.3	109/3.3	101/3.0		99/3.0	62/1.9	70/2.1	66/2.0	25/0.7	
Lunch is not allowed	0/0.0	0/0.0	40/7.0	7/1.7	6/1.0		0/0.0	0/0.0	24/0.7	14/0.4	15/0.4		17/0.5	13/0.4	10/0.3	8/0.2	5/0.1	
Dinner is not allowed	0/0.0	0/0.0	133/4.0	22/0.7	16/0.5		0/0.0	0/0.0	63/1.9	51/1.5	57/1.7		44/1.3	33/1.0	49/1.5	37/1.1	8/0.2	
Not at all	0/0.0	0/0.0	50/1.5	9/0.3	20/0.6		0/0.0	0/0.0	20/0.6	22/0.7	37/1.1		23/0.7	13/0.4	15/0.4	16/0.5	12/0.4	
Working years						**0.04**						1.32						0.32
≤ 1	0/0.0	0/0.0	115/3.4	15/0.4	15/0.4		0/0.0	0/0.0	48/1.4	56/1.7	41/1.2		47/1.4	39/1.2	31/0.9	20/0.6	8/0.2	
2–5	0/0.0	0/0.0	892/26.6	116/3.5	109/3.3		0/0.0	0/0.0	473/14.1	345/10.3	299/8.9		449/13.4	227/6.8	214/6.4	163/4.9	64/1.9	
6–10	0/0.0	0/0.0	838/25.0	103/3.1	102/3.0		0/0.0	0/0.0	456/13.6	324/9.7	263/7.9		427/12.8	215/6.4	197/5.9	144/4.3	60/1.8	
≥ 11	0/0.0	0/0.0	842/25.1	97/2.9	105/3.1		0/0.0	0/0.0	457/13.6	314/9.4	273/8.2		423/12.6	207/6.2	212/6.3	149/4.4	53/1.6	
Monthly income						22.85***						9.21***						12.00***
≤ 1000	0/0.0	0/0.0	10/0.3	4/0.1	15/0.4		0/0.0	0/0.0	6/0.2	1/0.1	22/0.7		5/0.1	3/0.1	4/0.1	5/0.1	12/0.4	
1001–2999	0/0.0	0/0.0	51/1.5	8/0.2	13/0.4		0/0.0	0/0.0	23/0.7	24/0.7	25/0.7		23/0.7	18/0.5	16/0.5	9/0.3	6/0.2	
3000–4999	0/0.0	0/0.0	921/27.5	107/3.2	133/4.0		0/0.0	0/0.0	503/15.0	352/10.5	306/9.1		499/14.9	242/7.2	201/6.0	155/4.6	64/1.9	
≥ 5000	0/0.0	0/0.0	1705/50.9	212/6.3	170/5.1		0/0.0	0/0.0	902/26.9	662/19.8	523/15.6		819/24.5	425/12.7	433/12.9	307/9.2	103/3.1	
Job satisfaction	——	——	22.4 ± 4.3	20.5 ± 3.7	20.3 ± 4.5	3.03***	——	——	23.2 ± 4.5	21.6 ± 3.9	20.7 ± 4.1	3.63***	23.3 ± 4.5	22.0 ± 3.8	21.0 ± 3.9	20.5 ± 3.7	20.4 ± 4.7	4.06***

Note: *P<0.05;**P<0.01<***P<0.001

The results of a single analysis showed that the years worked had no relation with the anxiety, depression, and stress symptoms of garden workers. Age and marital status were not related to anxiety symptoms among the garden workers. Based on the regression of orderly multi-classification logistics, gender, whether they could eat three meals on time, monthly income, and job satisfaction were the influencing factors affecting stress, anxiety, and depression symptoms in garden workers. (Tables 3, 4 and 5).

**Table 3:** Analysis of influencing factors of garden workers' stress

Variables	B	S.E.	Wald χ^2^	*P*	OR	95%CI	
Stress							
Normal	-4.035	0.906	19.846	<0.001	0.018	-5.811	-2.260
Mild	-3.124	0.905	11.922	0.001	0.044	-4.898	-1.351
Moderate	-2.076	0.904	5.274	0.022	0.125	-3.848	-0.304
Severe	-0.562	0.905	0.386	0.534	0.570	-2.336	1.212
Gender							
Male	-0.369	0.066	31.688	**<0.001**	0.691	-0.498	-0.241
Female	Ref						
Age							
≤ 30	0.371	0.619	0.360	0.549	1.450	-0.843	1.585
31–40	0.319	0.617	0.267	0.605	1.375	-0.890	1.527
41–50	0.116	0.618	0.035	0.851	1.123	-1.095	1.327
51–60	0.199	0.620	0.103	0.748	1.220	-1.017	1.415
≥ 60	Ref						
Marital status							
Married	-0.382	0.607	0.396	0.529	0.683	-1.571	0.808
Single	-0.270	0.613	0.194	0.660	0.763	-1.472	0.932
Divorce	-0.087	0.635	0.019	0.892	0.917	-1.331	1.157
Widowed	Ref						
Three meals on time							
All	-0.790	0.206	14.745	**<0.001**	0.454	-1.193	-0.387
Breakfast is not allowed	-0.292	0.226	1.658	0.198	0.747	-0.735	0.152
Lunch is not allowed	-0.322	0.320	1.012	0.314	0.725	-0.948	0.305
Dinner is not allowed	-0.255	0.245	1.084	0.298	0.775	-0.736	0.225
Not at all	Ref						
Monthly income							
≤ 1000	1.874	0.346	29.315	**<0.001**	6.516	1.196	2.553
1001–2999	0.217	0.219	0.989	0.320	1.243	-0.211	0.646
3000–4999	-0.093	0.070	1.780	0.182	0.911	-0.229	0.044
≥ 5000	Ref						
Job satisfaction	-0.119	0.008	233.481	**<0.001**	0.888	-0.134	-0.103

**Table 4:** Analysis of influencing factors of garden workers’ anxiety

Variables	B	S.E.	Wald χ^2^	*P*	OR	95%CI	
Anxiety							
Moderate	-3.536	0.280	159.944	<0.001	0.029	-4.083	-2.988
Severe	-2.123	0.275	59.544	<0.001	0.120	-2.662	-1.584
Gender							
Male	-0.132	0.066	3.960	**0.047**	0.876	-0.261	-0.002
Female	Ref						
Three meals on time							
All	-0.893	0.218	16.766	**<0.001**	0.409	-1.320	-0.465
Breakfast is not allowed	-0.524	0.238	4.847	**0.028**	0.592	-0.991	-0.058
Lunch is not allowed	-0.826	0.336	6.048	**0.014**	0.438	-1.485	-0.168
Dinner is not allowed	-0.527	0.258	4.161	**0.041**	0.590	-1.033	-0.021
Not at all	Ref						
Monthly income							
≤ 1000	1.937	0.419	21.335	**<0.001**	6.938	1.115	2.759
1001–2999	0.480	0.224	4.600	**0.032**	1.616	0.041	0.918
3000–4999	0.061	0.069	0.772	0.380	1.063	-0.075	0.197
≥ 5000	Ref						
Job satisfaction	-0.108	0.008	186.892	**<0.001**	0.898	-0.124	-0.093

**Table 5:** Analysis of influencing factors of garden workers' depression

Variables	B	S.E.	Wald χ^2^	*P*	OR	95%CI	
Depression							
Moderate	-2.025	1.365	2.2	0.138	0.132	-4.701	0.651
Severe	-1.159	1.365	0.721	0.396	0.314	-3.835	1.516
Gender							
Male	-0.559	0.092	36.914	**<0.001**	0.572	-0.74	-0.379
Female	Ref						
Age							
≤ 30	0.374	1.113	0.113	0.737	1.454	-1.807	2.555
31–40	0.404	1.11	0.132	0.716	1.498	-1.771	2.579
41–50	0.376	1.111	0.115	0.735	1.456	-1.802	2.554
51–60	0.735	1.113	0.436	0.509	2.085	-1.446	2.917
≥ 60	Ref						
Marital status							
Married	-0.437	0.721	0.368	0.544	0.646	-1.851	0.976
Single	-0.03	0.731	0.002	0.967	0.970	-1.464	1.404
Divorce	-0.008	0.760	0.000	0.992	0.992	-1.498	1.482
Widowed	Ref						
Three meals on time							
All	-1.068	0.242	19.501	**<0.001**	0.344	-1.541	-0.594
Breakfast is not allowed	-0.564	0.27	4.357	**0.037**	0.569	-1.093	-0.034
Lunch is not allowed	-0.695	0.402	2.982	0.084	0.499	-1.484	0.094
Dinner is not allowed	-0.949	0.304	9.736	**0.002**	0.387	-1.546	-0.353
Not at all	Ref						
Monthly income							
≤ 1000	2.03	0.378	28.881	**<0.001**	7.614	1.29	2.771
1001–2999	0.622	0.268	5.369	**0.02**	1.863	0.096	1.147
3000–4999	0.144	0.096	2.214	0.137	1.155	-0.046	0.333
≥ 5000	Ref						
Job satisfaction	-0.111	0.011	98.474	**<0.001**	0.895	-0.133	-0.089

## Discussion

The results of this study showed that, compared to other groups, the anxiety and depression symptoms of Chinese garden workers are generally high [43–45]. With the rapid development of Chinese society, the requirements of cities for horticulture are constantly rising, and an increasing number of cities are focusing on improving their liveability through greening. It was in this context that the National Botanical Garden workers. The vast majority of China’s garden workers are rural migrant workers who are urban migrants, and this portion of the population bears the responsibility of providing for the family and needs this job to maintain the normal operation of the family [46]. Most migrant workers are young people whose income levels, social security, welfare benefits, and living conditions are far lower than those of the urban residents. This undoubtedly aggravates the anxiety and depression in this group. Attention to this group should be intensified in the day-to-day functioning of society and should not be ignored.

An interesting phenomenon was found in the results: although garden workers have lower levels of stress, they have higher levels of anxiety and depression. Previous research found that stress is a predictive factor for anxiety and depression, and individuals with higher levels of stress have higher levels of anxiety and depression [47]. However, stress is only one of the risks that cause individual anxiety and depression, and other factors (e.g. anxiety and social support) have a significant impact on an individual’s level of anxiety and depression [47, 48]. Stress, anxiety, and depression are interrelated and independent. Therefore, in management, more attention should be paid to the impact of factors other than stress on anxiety and depression among horticultural workers.

This study found that gender is a contributing factor to the stress, anxiety, and depressive states of garden workers. This result confirms the results of previous studies [49]. The findings of Carlo Faravelli et al. showed that the lifetime prevalence of affective disorders is higher in women than in men [50]. The National Institute of Mental Health reports that the lifetime prevalence of anxiety disorders in women is 60% higher than in men [51, 52]. In traditional societies, women were endowed with passivity, submissiveness, and dependence, and were obliged to take on the label of relentless care for others and unpaid domestic and agricultural labour. This gender gap affects the power and control of men and women over these socioeconomic determinants; their access to resources; status, role, choice, and treatment in society. This may enhance the subordinate status of female gardeners who are more likely to work in unsafe and low-status jobs, leading this group to experience higher levels of negative life events, more chronic stressors, and less social support, resulting in anxiety and depression symptoms. Improving the balance between gender roles and obligations, pay equity, poverty reduction, and a renewed focus on sustaining social capital will further correct the gender gap in mental health and lead to improvements in women’s mental health [53].

Furthermore, the results showed that having three meals on time is also an important factor affecting the mental health of garden workers. This finding has not been previously reported. Analyzing the reasons for this, the demand for musculoskeletals in horticulture is enormous, with gardeners requiring the operation of large hand tools such as shovels and hoes, as well as power equipment such as lawn mowers, hedge trimmers, leaf blowers, and wood chippers [7]. However, in daily work, because of the special nature of work, mobile operations are required, and some garden workers are in the process of mobile operations; they cannot eat at fixed places but need to be delivered by relevant personnel to deliver box lunches. As a result, garden workers are unable to eat on time. Being unable to eat on time for a long time causes them to be weak in their daily work, consume their existing energy excessively, affect gardeners’ negative evaluation of the working environment, and produce negative emotions.

The results of this study also show that monthly income is an important factor affecting the physical and mental health of garden workers. Previous studies found a strong correlation between income and various health outcomes [54, 55], those employees with higher levels of economic stress show higher levels of absenteeism, which simultaneously contributes to a decrease in innovative behaviours [56]. Income affects people’s health by directly influencing the material conditions an individual needs for survival as well as by influencing an individual’s chances of social participation and control of the living environment [57]. Most garden workers are manual workers and have greater labour intensity, and if lower income is not proportional to the labour intensity borne by the body, it leads to psychological imbalance. In addition, most workers have family responsibilities, and their lower incomes make it difficult for them to provide more support to themselves and their families, which in turn leads to negative mental health. Therefore, it is necessary to improve the mental health of horticultural workers by providing them with an income that can ensure their quality of life.

Moreover, job satisfaction is also an important factor affecting the mental health of garden workers. Previous studies have reported similar findings [44, 58, 59]. The association between job satisfaction and psychological distress is stronger than that with physical illness [58]. Several people spend a significant portion of their time at work when awake. If their work fails to provide sufficient personal satisfaction, they are likely to feel unhappy or dissatisfied for a long period of time each working day, creating an increased risk of decreased overall mood and sense of self-worth and ultimately leading to depression and/or anxiety [58]. Work adaptation theory suggests that satisfaction occurs when an organisation can match its employees’ values, interests, and needs of its employees [60]. Most garden workers are manual workers, and at the bottom of society, gender discrimination, inability to eat normally, and low-income factors may not meet the personal needs of garden workers, triggering gardeners’ dissatisfaction with their work and resulting in negative mental health problems. Faragher et al. argue that risk assessments of workplace stress should attempt to identify the aspects of work that cause employees to be most dissatisfied as these can also contribute to elevated stress levels [58]. After meaningful consultations with employees, work practices should be appropriately changed, and the impact of these practices on stress levels and job satisfaction should be measured. This will have the greatest benefits for employee mental health through its impact on job satisfaction (particularly the reduction in the degree of burnout/emotional exhaustion) and a beneficial knock-on effect on organizational health [58].

Injuries and illnesses cost employers about $60 billion a year. The costs of different types of injuries vary, but the cost of enterprise overwork is about US $13.79 billion per year [61]. Negative emotions such as stress, anxiety, and depression can negatively impact an individual’s physical health [62], and mitigating the impact of such emotions and improving the mental health of individuals is a problem that managers need to think about. In daily work, enterprises and government departments should continuously improve the working environment of garden workers, provide salaries that match their positions, improve the recognition and job satisfaction of garden workers, pay attention to changes in individual emotions, and implement interventions to reduce the impact of negative emotions on personal health to improve the personal health of garden workers and maintain their physical and mental health.

The results of this study provide a reference for unions in the region to intervene effectively in the mental health of garden workers, formulate relevant policies and measures, improve the working environment, and health. Furthermore, the results of this study can provide a reference for other developing countries and developed countries to conduct research on the mental health of garden workers in their respective countries.

### Limitations

This study has the following limitations. First, the study was conducted in the context of the COVID-19 pandemic. Garden workers’ mental health may have been affected by this factor, resulting in higher anxiety and depression scores. After the end of the COVID-19 pandemic, or in future research, this factor will be controlled to better understand the physical and mental health status of Chinese garden workers. Second, the cross-sectional study design may have led to an inability to determine causality. Third, this study did not consider the impact of regional, social, or cultural background factors on the physical and mental health of the garden workers. Future research should focus more on this issue and clarify the mechanisms by which social and cultural factors affect the physical and mental health of garden workers.

## Conclusions

Chinese garden workers are more stressed, anxious, and depressed. Gender, whether they can eat three meals on time, monthly income, and job satisfaction are important factors affecting their mental health. Enterprises and the government should address this problem by continuously improving the working environment of garden workers, providing salaries that match their positions, and increasing the recognition and job satisfaction of garden workers to reduce the impact of negative emotions on personal health, thereby improving personal health level of garden workers and maintaining physical and mental health.

## Data Availability

The original contributions presented in the study are included in the article, further inquiries can be directed to the corresponding authors.

## Abbreviations

COVID-19: Coronavirus Disease 2019
PTSD: Post-traumatic stress disorder
HCP: Healthcare providers
DASS: Depression Anxiety Stress Scale
